# Cost of Pacing in Pediatric Patients With Postoperative Heart Block After Congenital Heart Surgery

**DOI:** 10.1001/jamanetworkopen.2023.41174

**Published:** 2023-11-03

**Authors:** Abhijit Mondal, Minkyoung Yoo, Stephanie Tuttle, Douglas Mah, Richard Nelson, Frank B. Sachse, Robert Hitchcock, Aditya K. Kaza

**Affiliations:** 1 Department of Cardiac Surgery, Boston Children’s Hospital, Boston, Massachusetts; 2 Department of Surgery, Harvard Medical School, Boston, Massachusetts; 3 Division of Epidemiology, University of Utah, Salt Lake City; 4 Department of Cardiology, Boston Children’s Hospital, Boston, Massachusetts; 5 Department of Pediatrics, Harvard Medical School, Boston, Massachusetts; 6 Department of Biomedical Engineering, University of Utah, Salt Lake City

## Abstract

**Importance:**

Surgical correction of congenital heart defects (CHDs) has improved the lifespan and quality of life of pediatric patients. The number of congenital heart surgeries (CHSs) in children has grown continuously since the 1960s. This growth has been accompanied by a rise in the incidence of postoperative heart block requiring permanent pacemaker (PPM) implantation.

**Objective:**

To assess the trends in permanent pacing after CHS and estimate the economic burden to patients and their families after PPM implantation.

**Design, Setting, and Participants:**

In this economic evaluation study, procedure- and diagnosis-specific codes within a single-institution database were used to identify patients with postoperative heart block after CHS between January 1, 1960, and December 31, 2018. Patients younger than 4 years with postoperative PPM implantation were selected, and up to 20-year follow-up data were used for cost analysis based on mean hospital event charges and length of stay (LOS) data. Data were analyzed from January 1, 2020, to November 30, 2022.

**Exposure:**

Implantation of PPM after CHS in pediatric patients.

**Main Outcomes and Measures:**

Annual trends in CHS and postoperative PPM implantations were assessed. Direct and indirect costs associated with managing conduction health for the 20 years after PPM implantation were estimated using Markov model simulation and patient follow-up data.

**Results:**

Of the 28 225 patients who underwent CHS, 968 (437 female [45.1%] and 531 male [54.9%]; 468 patients aged <4 years) received a PPM due to postoperative heart block. The rate of CHS and postoperative PPM implantations increased by 2.2% and 7.2% per year between 1960 and 2018, respectively. In pediatric patients younger than 4 years with PPM implantation, the mean (SD) 20-year estimated direct and indirect costs from Markov model simulations were $180 664 ($32 662) and $15 939 ($1916), respectively. Using follow-up data of selected patients with clinical courses involving 1 or more complication events, the mean (SD) direct and indirect costs were $472 774 ($212 095) and $36 429 ($16 706), respectively.

**Conclusions and Relevance:**

In this economic evaluation study, the cost of PPM implantation in pediatric patients was found to accumulate over the lifespan. This cost may represent not only a substantial financial burden but also a health care burden to patient families. Reducing the incidence of PPM implantation should be a focused goal of CHS.

## Key Points

**Question:**

What is the estimated economic burden of permanent pacemaker (PPM) implantation and management of pacing in pediatric patients after congenital heart surgery (CHS)?

**Findings:**

In this economic evaluation study using single-institution data of 28 225 patients who underwent CHS, the estimated 20-year mean direct and indirect costs for PPM implantation and management per patient were $180 664 and $15 939, respectively. Complications, though rare, increased these costs to $472 774 and $36 429, respectively.

**Meaning:**

The findings of this study suggest that PPM implantation due to postoperative heart block in pediatric patients imposes a substantial financial burden on patients and their families; thus, reducing the incidence of PPM implantation should be a focused goal of CHS.

## Teaser

This economic evaluation study assesses the trends in permanent pacemaker implantation after pediatric congenital heart surgery and estimates the 20-year financial burden to patients and their families.

## Introduction

Since the first palliative and intracardiac surgeries for correction of congenital heart defects (CHDs) in the 1930s and 1950s,^1^ surgeons have been concerned about trauma to the cardiac conduction system that may result in heart block. Despite the success and improvement of techniques in these early years, approximately 1 in 10 patients developed postoperative heart block due to damage to the cardiac conduction system during surgery.^2^

The incidence of postoperative heart block varies depending on the type and complexity of congenital heart surgery (CHS).^3,4^ Early pacemaker technology was motivated and developed to treat heart blocks associated with CHS. Despite this motivation, commercial development of rhythm management technology and guidelines for clinical practice focused on adults, since the market was much larger. The first clinical practice guidelines for pacemaker implantation in pediatric patients were published in 1998 and revised in 2002.^5^ In pediatric patients, the permanent pacemaker (PPM) must be implanted to accommodate both patient size and subsequent growth.^6,7,8^ For patients weighing less than 10 to 15 kg, epicardial leads are favored, as endocardial leads have procedural challenges and an increased risk of venous occlusion and valve damage.^9,10^ However, epicardial leads generally result in more complications. In addition to specific guidelines, technology has also improved for pediatric patients^7^ and advanced steadily over the decades.^11,12^

There is a considerable burden associated with PPM implantation in pediatric patients. This burden affects the patients and the patients’ families. Previous reports on this burden included evaluation of the medical costs associated with a PPM and implantable cardioverter-defibrillator (ICD) procedures,^13^ indirect costs for families living with a child with a CHD,^14^ resource utilization during emergency department visits for children with CHDs,^15^ and examination of self-competence and psychosocial functioning of pediatric patients with PPMs.^16,17^ None of these reviews examined the lifetime burden of PPM implantation in pediatric patients.

We aimed to estimate the societal burden caused by PPM implantation in pediatric patients with acquired heart block. Given the technological advancements of intraoperative techniques for identifying conduction tissues,^18^ iatrogenic heart blocks could become much less common. This is the first study to our knowledge to assess the direct and indirect medical costs associated with PPMs incurred over 20 years after implantation. We also present the trends in CHS and postoperative PPM implantations in a single specialty hospital.

## Methods

### Data Source and Collection

**Figure 1.**
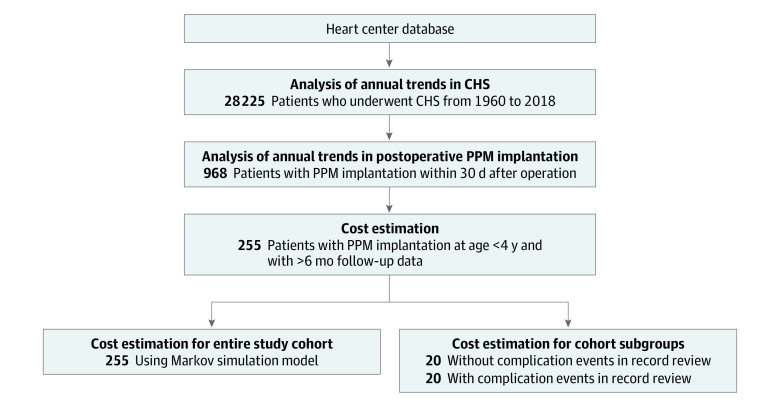
Outline of Research Methods and Results The flowchart describes the basic research method steps and presented results. An economic evaluation was performed using 2 approaches: (1) estimate of the cost of the entire study cohort and (2) estimate of the costs of each subgroup of patients with and without clinical course complications. CHS indicates congenital heart surgery; PPM, permanent pacemaker.

In this economic evaluation study, we collected patients’ medical procedure data from the Boston Children’s Hospital’s Heart Center database. Figure 1 summarizes the study outline and the patient cohort selection criteria. For each deidentified patient procedure (inpatient or outpatient), data on patient’s age, procedure dates, length of stay (LOS), and attending surgeons were collected. Race and ethnicity data were not collected because this information was unavailable for a major portion of the study period. The study protocol was conducted per Boston Children’s Hospital human subject committee guidelines and approved by the institutional review board (IRB-P00030677) with a waiver of informed consent because the proposed use of the data presented no risk to the privacy of individuals. The research could not practicably be conducted without the waiver of informed consent and authorization. The study followed the Consolidated Health Economic Evaluation Reporting Standards (CHEERS) reporting guideline.

### Patient and Procedure Data Classification

Patients with PPM implantations between January 1, 1960, and December 31, 2018, were first identified using institutional PPM-associated procedure codes, including single-chamber, dual-chamber, and biventricular pacing systems. Patients with ICDs were excluded. Within these patients, postoperative PPM was identified if PPM implantation was within 30 days of CHS.

For the postoperative PPM cost estimation analysis, patients younger than 4 years at PPM implantation with at least 6 months of available follow-up data were selected. Most surgical correction of congenital defects are performed before age 4 years.^19^ Procedure codes for major PPM-related events, including PPM implantation, generator change or battery depletion, PPM malfunction requiring lead and generator replacement, and PPM infection, were used to identify major PPM-associated clinical events. Hospital visits associated with monitoring and/or the treating patient’s cardiac conduction system were included as minor events and comprised in-clinic visits, in-clinic device checks, standard and Holter electrocardiograms, electrophysiology (EP) catheterization procedures (EP study or radiofrequency ablation), and cardiac rehabilitation. Permanent pacemaker malfunctions, such as oversensing, undersensing, output failure, or failure to capture, can be resolved by reprogramming the generator or adjusting the leads. Hospital events associated with such PPM malfunctions were subsumed as minor events like EP catheterization and in-clinic device checks.

### Trends in CHS and Postoperative PPM Implantation

Using procedure codes and event dates, CHSs and postoperative PPM implantations occurring between 1960 and 2018 were identified. Each event was binned to year of surgery and grouped based on patient age (<4 years and ≥4 years). The eMethods in Supplement 1 provide more details.

### Cost Estimation Using Markov Simulation Model of a Patient

A Markov simulation model was developed for patients who underwent CHS with postoperative PPM implantation.^20^ The model (eFigure 2 in Supplement 1), with a cycle length of 1 year, was evaluated from a hospital perspective over a 20-year time horizon on a modeled population of 10 000 hypothetical patients. The movement of hypothetical patients through the model was governed by probability input parameters (Table). In the Markov model, patients entered the model by having a PPM implanted after CHS before age 4 years. After the implantation, patients could present for follow-up with a major or minor PPM-related event (eFigure 2 in Supplement 1). Patients could experience each event multiple times during a 20-year time horizon. Outcomes were discounted by 3% annually. The model was programmed using TreeAge Pro 2018, version 2.1 (TreeAge Software Inc). The eMethods in Supplement 1 provide more details.

**Table.** Markov Model Input Parameters: Probabilities, Length of Stay (LOS), and Costs of Permanent Pacemaker (PPM)–Related Procedures and Complications

	Probability^a^	Mean (SD)		
		LOS, d^a^	Cost, 2018 USD	
			Direct^b^	Indirect^c^
PPM implantation	NA	13.005 (13.166)	108 052 (109 053)	7338 (7429)
Major complication (annual %)				
PPM malfunction requiring lead and generator replacement	2.0	3.156 (3.586)	52 720 (52 720)	1792 (2035)
Generator replacement	11.2	0.322 (1.039)	41 797 (11 050)	355 (600)
PPM infection	0.6	12.679 (15.573)	91 653 (112 100)	7154 (8784)
Minor event (annual frequency)				
In-clinic consultations or visits	1.5	0.125 (NA)	219 (NA)	136 (NA)
In-clinic device check	0.9	0.125 (NA)	463 (NA)	136 (NA)
ECG	1.8	0.125 (NA)	289 (NA)	136 (NA)
Holter	0.1	0.125 (NA)	963 (NA)	136 (NA)
EP catheterization procedure	0.1	1.000 (NA)	5894 (NA)	578 (NA)
Annual discount rate, %	3.0	NA		

Abbreviations: ECG, electrocardiogram; EP, electrophysiology; NA, not applicable; USD, US dollars.

^a^ Calculated using data from Boston Children’s Hospital Heart Center database.

^b^ Calculated using data from eTable 1 in Supplement 1.

^c^ Calculated using data from eTable 2 in Supplement 1.

### Cost Estimation of Clinical Courses With and Without Complication Using Follow-Up Data

Due to limited follow-up data, 20-year costs were first estimated from selected patients who had (1) a clinical course without complication (n = 20) and (2) a clinical course with complication (n = 20). Patients with a clinical course with complications experienced 1 or more PPM-associated malfunction (requiring generator and lead replacement) and/or infection events. For each selected patient, a timeline of all relevant clinical events starting from postoperative PPM implantation was created. Direct and indirect costs were calculated using estimated rates (eTables 1 and 2 in Supplement 1) and LOS. The eMethods in Supplement 1 provide more details.

### Statistical Analysis

We performed the data analysis from January 1, 2020, to November 30, 2022. Linear regression (LR) analysis and a Poisson generalized linear model (GLM) were used to assess statistical significance levels (eMethods in Supplement 1). A 2-sided *P* < .05 was considered significant. The analyses were performed using and Excel 365, version 1912 (Microsoft Corporation) and TreeAge Pro 2018, version 2.1.

## Results

The study included 28 225 patients who underwent 38 723 CHSs by 25 surgeons. Of these patients, 968 (437 female [45.1%] and 531 male [54.9%]) received a PPM within 30 days of their CHS. Within this group, 468 patients younger than 4 years underwent PPM implantation, and 255 of these patients had at least 6 months of available follow-up data.

### Trends in Cardiac Surgeries

**Figure 2.**
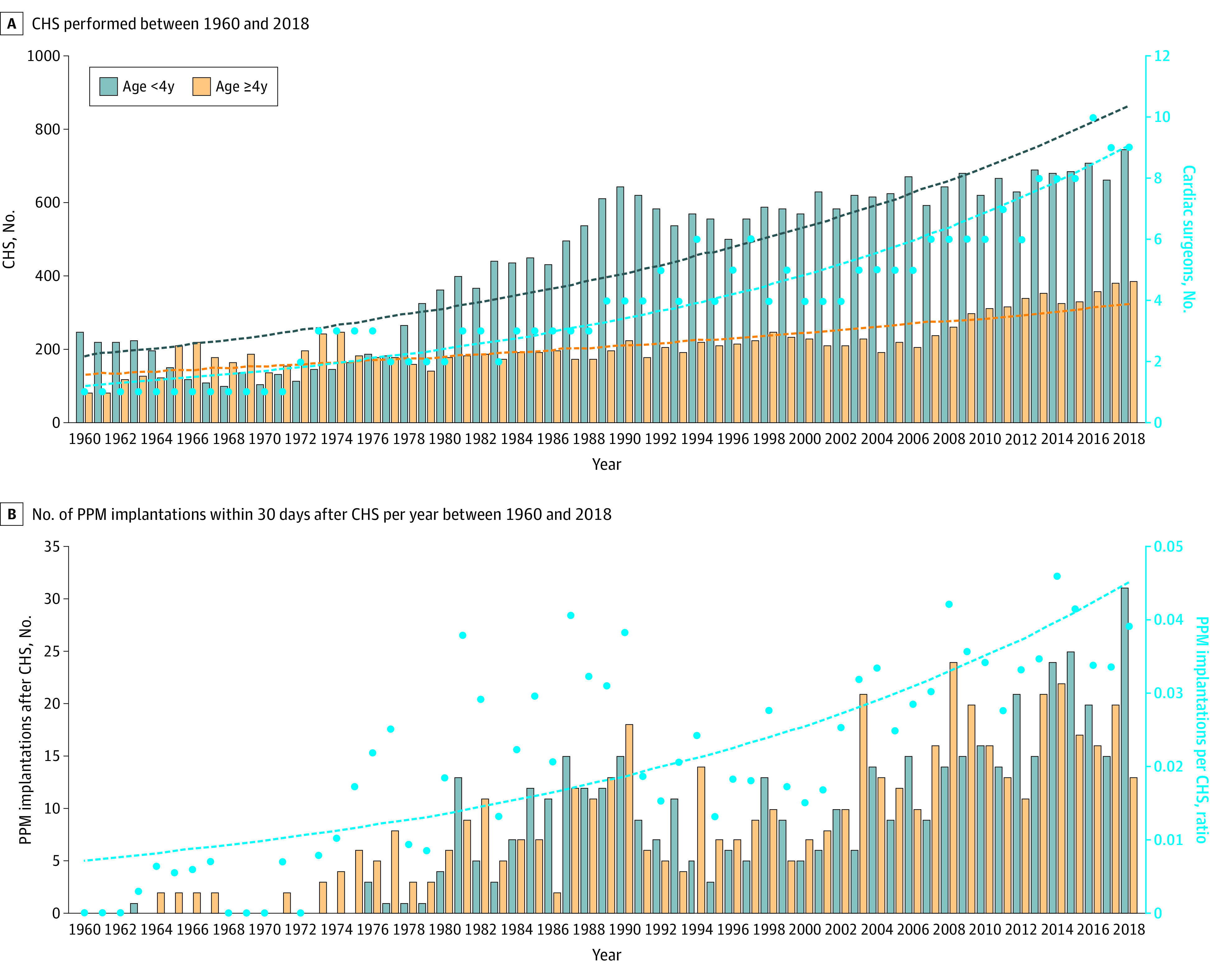
Trends in Congenital Heart Surgery (CHS) and Postoperative Permanent Pacemaker (PPM) Implantations at Boston Children’s Hospital (A) The number of CHSs performed in patients aged younger than 4 years (blue dashed curve) (generalized linear model [GLM]: β = 0.027; *P* < .001) increased at a faster rate than those performed in patients aged 4 years or older (orange dashed curve) (GLM: β = 0.015; *P* < .001). The number of surgeons (blue dots) performing CHSs also increased (black dashed curve) (GLM: β = 0.035; *P* < .001). (B) The total number of postoperative PPM implantations per CHS (blue dots) also increased (blue dashed curve) (GLM: β = 0.031; *P* = .59).

Our analysis of patient records at Boston Children’s Hospital from 1960 to 2018 showed a greater than 3-fold increase in CHSs (Figure 2A). The overall compound annual growth rate (CAGR) from 1960 to 2018 was 2.2%. The surgical team expanded over time, with the number of surgeons increasing from 1 to 10 in the same period (GLM: β = 0.035; *P* < .001) . The number of surgeries performed in patients younger than 4 years (GLM: β = 0.027, *P* < .001) increased at a higher rate than those performed in patients aged 4 years or older (GLM: β = 0.015; *P* < .001). Over the 58-year recorded history, 2 decades account for most of the growth: 1980 to 1990 (CAGR, 4.8%) and 2008 to 2018 (CAGR, 2.3%).

### Trends in Postoperative PPM Implantation

The number of postoperative PPM implantations increased, while implantations per CHS rose until 1976 and fluctuated until 2018 (Figure 2B). While the general trend shows a rise (GLM: β = 0.031; *P* = .59), there was a notable deviation from this trend between 1975 and 1990, with a higher number of PPM implantations per CHS. The CAGR of total postoperative PPM implantations from 1963 to 2018 was 7.2%. When the PPM implantations were normalized with respect to the number of surgeries performed, the CAGR was reduced to 4.9% over the same period. The CAGR for PPM implantations in patients younger than 4 years in the same period was 6.5% compared with 3.5% for patients 4 years or older at implantation.

### Direct and Indirect Medical Cost Estimation

The mean (SD) direct and indirect costs incurred during the initial PPM implantation were $108 052 ($109 053) and $7338 ($7429), respectively. The mean (SD) direct and indirect costs associated with PPM infection were $91 653 ($112 100) and $7154 ($8784), respectively, and for PPM malfunction, $52 720 ($52 720) and $1792 ($2035), respectively (Table). Regression analysis of hospital billing data revealed that the cost of PPM implantation (LR: *R*^2^ = 0.838; *P* < .001) and cost of infection (LR: *R*^2^ = 0.864; *P* < .001) strongly depended on LOS (eFigure 1A and D in Supplement 1). The cost of a PPM malfunction event (LR: *R*^2^ = 0.23; *P* < .050) was weakly dependent on LOS, while that of battery depletion or generator change (LR: *R*^2^ = 0.007; *P* = .72) was independent of LOS (eFigure 1B and C in Supplement 1).

### Markov Simulation Model for Total Cost Estimation

For the selected patient cohort, the annual probabilities of a PPM malfunction requiring lead and generator replacement and infection were 2.0% and 0.6%, respectively. For an event requiring generator change, the probability was 11.2%. The corresponding LOS was low to moderate for malfunction (mean [SD], 3.156 [3.586] days), moderate to high for infection (mean [SD], 12.679 [15.573] days), and very low for generator change (mean [SD], 0.322 [1.039] days). The Table summarizes these results used as input parameters for the Markov model.

In the base case analysis using the complication probabilities, LOS (Table), and direct and indirect costs for complications (eTables 1 and 2 in Supplement 1), the mean (SD) estimated 20-year direct cost per patient for postoperative PPM implantation was $180 664 ($32 662), and the indirect cost was $15 939 ($1916). The results from probabilistic sensitivity analysis varying input parameters remained consistent with the base case analysis with larger SDs (mean [SD] 20-year direct cost per patient, $182 829 [$115 770]; mean [SD] 20-year indirect cost per patient, $16 303 [$7676]).

### Twenty-Year Cost of Chronic Pacing of Different Clinical Courses Using Follow-Up Data

**Figure 3.**
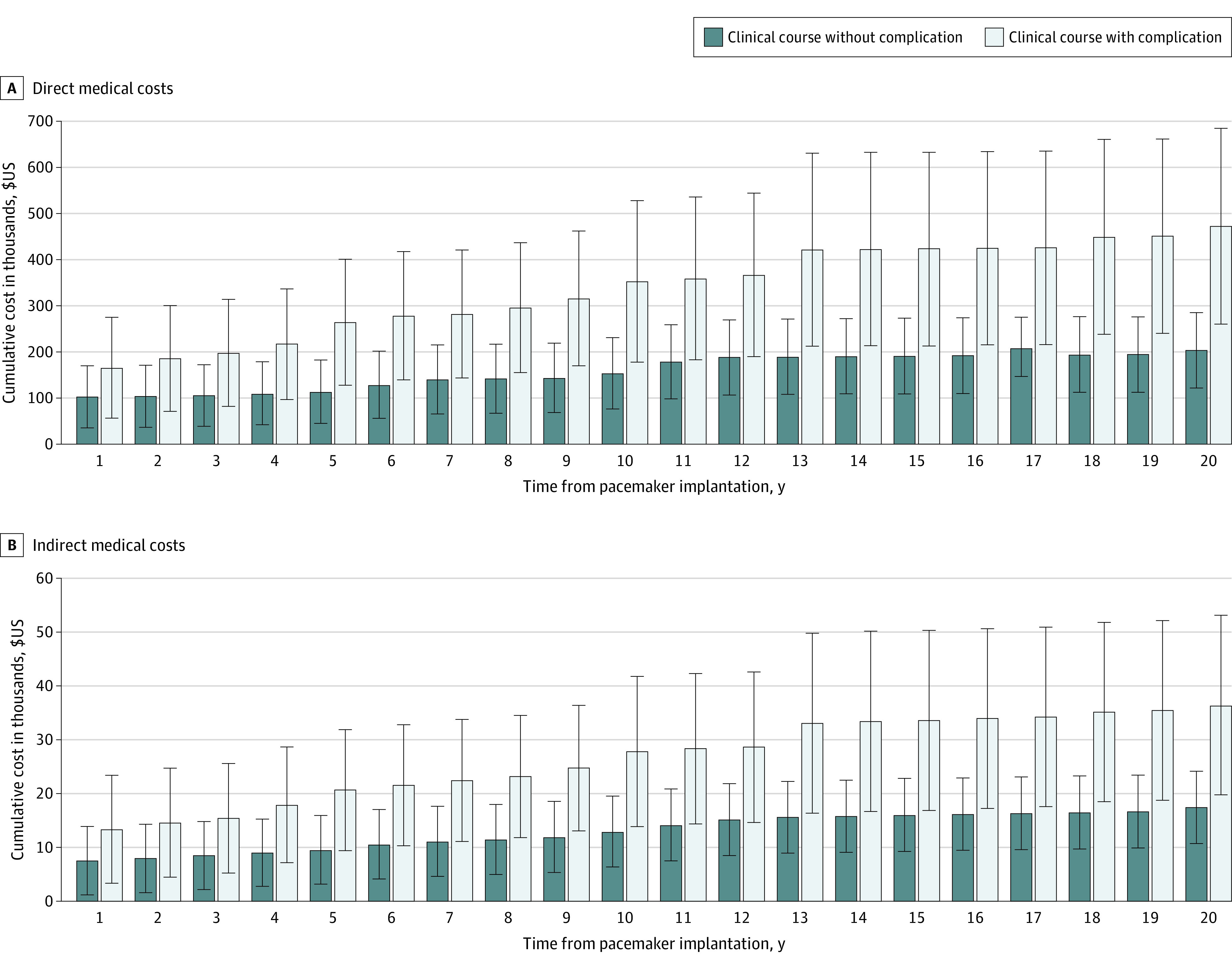
Estimated 20-Year Cumulative Costs of Chronic Pacing in Pediatric Patients Cumulative direct medical costs are associated with permanent pacemaker–related procedures and patient cardiac conduction system monitoring, and cumulative indirect costs include those to the patient and their family to accompany them for medical treatment and care. Both direct medical and indirect costs rose nonlinearly over the 20 years, with approximately 35% to 50% of the total cost spent in the first year after implantation. Hospital event data were collected from 20 patients for each cohort. The sample size for each year in each group varies based on available patient follow-up data.

The cohort of selected patients with no complications had a higher rate of complex lesions than the overall cohort (80% vs 52%) (eFigure 3 in Supplement 1). For the clinical course during and after CHSs without complication, cumulative costs from years 10 to 19 were extrapolated to estimate the 20-year direct (LR: *R*^2^ = 0.568; *P* < .050) and indirect (LR: *R*^2^ = 0.823; *P* < .001) costs. Figure 3 summarizes the results. The mean (SD) 20-year direct cost for the clinical course without complication was $203 767 ($81 831), and the direct cost for patients with 1 or more complication events was $472 774 ($212 095). The mean (SD) 20-year indirect cost for the clinical course without complication was $17 478 ($6720), and the indirect cost for the clinical course with complications was $36 429 ($16 706).

## Discussion

In this economic evaluation study, we used hospital procedure charge data, the institutional clinical database, and a probability model to gain a better perspective on the incidence, prevalence, and costs associated with postoperative PPM implantation in pediatric patients. We estimated the lifetime fixed and variable costs of PPM implantation, as well as complication rates and the additional costs associated with a clinical course involving PPM-associated complications. Our analysis showed that the number of pediatric cardiac procedures increased annually (Figure 2A), with the number of PPMs implanted also increasing (Figure 2B). The incidence of PPM implantation did not decrease despite advances in training, awareness of complications, and technological advances. We also found that the direct and indirect costs associated with pediatric PPM implantation were high for both the families and the health care system (Figure 3).

The majority of patients with CHS treated at Boston Children’s Hospital came from the 6 New England states. This region experienced an annual average population growth of 0.6% over the selected 58-year period.^21^ This simple perspective indicates that population growth was not solely responsible for the growth in CHS cases. Additional surgical volume can be attributed to referrals from outside the region. Surgical advances have also catalyzed the growth in CHSs, with both technology and training increasing confidence and allowing surgeons to routinely perform more complex procedures.^22,23^ Another factor in the increased number of surgeries is the use of staged surgical procedures. In these cases, a patient undergoes multiple surgeries over the first few years of life to repair more complex lesions. Each surgical stage accounts for a separate surgery. These types of surgical plans are critical for repairing the most complex lesions, such as congenitally corrected transposition, borderline left heart structures, unbalanced atrioventricular canals, and others. Complex cases have a higher risk of postoperative conduction anomalies.^4^ The most serious sequela of these conduction abnormalities is complete and chronic heart block, which necessitates the implantation of a PPM and could explain the higher-than-expected normalized PPM implantation rate between 1975 and 1990 (Figure 2B), when surgical techniques enabling the repair of complex defects were emerging.^24^ Thus, with the increased number of surgeries (Figure 2A) came a steady increase in PPM implantations (Figure 2B).

Considering the Markov model results, the mean (SD) direct cost of PPM implantation ($108 052 [$109 053]) is approximately 60% of the total 20-year cost ($180 664 [$32 662]), indicating that the majority of the cost was incurred in the first year after implantation, which may be partially due to the additional inpatient care needed for the first implant after CHS. The 20-year cost incurrence rate increased significantly if a major complication occurred. Our estimates show that a major PPM malfunction and infection event could increase the 20-year cost to 1.5 to 2.3 times and 3.2 to 4.3 times that of the normal clinical course, respectively (Figure 3). While the probability of infection was the least among complications, the cost of treatment can be high. Depending on the severity of the infection, a patient might receive outpatient treatment with intravenous antibiotic therapy or be hospitalized for an extended period if a change of generator or complete generator and lead replacement are required. In the case of a PPM malfunction requiring lead and generator replacement, the costs were even higher with longer LOS. In these cases, the EP team must first diagnose the source of device malfunction before determining the treatment course, which can be as simple as a standard generator check or could require the need for EP catheterization studies. The course could include the need for a new pacing site, change in pacing strategy, or a device upgrade, and the patient may have developed an arrhythmia that requires EP ablation or even an ICD implantation. Complications associated with PPMs when receiving inpatient treatment may require intensive care unit admission, which has a higher hospital charge.

The mean 20-year indirect cost is approximately one-tenth of the corresponding direct cost but directly incurred by the patient’s family and paid out of pocket (Figure 3). Our estimates were conservative, as we assumed 1 adult accompanying the child to the hospital and only considered the on-the-ground costs associated with patient LOS and hospital visits. Nonmedical out-of-pocket expenses^25^ of sibling care, homemaking, and incidental expenses were not considered. Even the conservative 20-year indirect cost estimates varied from 0.3 to 1.1 times the mean annual US household income of $61 372 in 2018^21^ (Figure 3B; eTable 2 in Supplement 1). High indirect costs incurred during the initial implantation (mean [SD], $7338 [$7429]) or complication (PPM malfunction, $1792 [$2035]; PPM infection, $7154 [$8784]) can financially strain the average household (Table).

The number of CHSs grew continuously since surgical repairs were initiated in the early 1960s. This growth was driven by the refinement of the procedures and an increase in the number of trained surgeons and qualified institutions. It also was driven by population growth. Some of the growth can be attributed to our ability to repair increasingly complex cardiac lesions. However, the unintended consequence of this growth in complex cases was the concomitant rise in the incidence of PPM implantation. Our study focused on the economic burden of PPM implantation in children, but there are social and psychological problems as well that could be associated with pediatric patients’ quality of life.^16,17,26^ Several technologies have been introduced to identify and avoid damage to the cardiac conduction system during surgery,^18^ but the incidence rate continues to creep up. We have developed advanced surgical technology to rescue children from previously fatal CHDs. We should now apply the same diligence to focus on this serious comorbidity and mitigate the unintended consequence of a lifetime of cardiac pacing.

### Limitations

This study has several limitations. It was performed at a single specialty hospital, which limits geographic generalization of costs. Despite this limitation, the study provides perspective and identifies potential drivers of direct and indirect costs. Another limitation is the decade-to-decade economic variability during the selected period between 1960 and 2018. Although a period-wise assessment would be insightful, it is not feasible with the limited number of patients and follow-up data from a single center.

For hospital event LOS, it was not feasible to delineate the exact LOS associated with the first PPM implantation from that associated with postoperative recovery and/or complications for all patients. Hence, LOS specifically associated with PPM implantation after the index surgery was approximated using the discharge date or the next procedure (surgery or catheter intervention) date.

While the Markov model results were from the larger patient cohort, the selected patient cohort analysis (Figure 3A and B) may provide a snapshot of costs for patients with complications. Ideally, we expected the model results from a larger cohort to be between the costs from the 2 subgroups, but the model results were approximately 10% lower than the selected, no-complication cohort. The smaller cohort of patients with an uncomplicated clinical course had a higher number of complex lesions (80% vs 52%) (eFigure 3 in Supplement 1). Patients with complex lesions may need more medical care and, therefore, may have higher postsurgical LOS and more hospital visits than those with simpler lesions. These complex lesion cases could explain the slightly higher mean cost in the smaller cohort of patients without complications. Nevertheless, the mean costs between the 2 methods are within 10% and 1 SD.

Our current analysis using the Markov model did not reflect the time-sensitive event probability because we defined the event probabilities by counting the number of events from the pulled data of all the cases during the follow-up periods. Furthermore, we did not conduct a cost-effectiveness analysis due to the lack of information in the literature on the utility value.

## Conclusions

In this economic evaluation study, we present conservative estimates of the financial costs of PPM implantation in pediatric patients after CHS that patients and their families could incur over the first 20 years after implantation. Though rare, PPM-associated major complication events are associated with high costs that could severely affect a family’s financial well-being. Defining these costs may offer a basis for innovation to further motivate the prevention of postoperative heart block after CHS.

## Notes

Supplement 1.
eMethods
**eTable 1.** Medical Procedure Costs **eTable 2.** Cost Considerations for Accompanying a Patient to the Hospital **eFigure 1.** Permanent Pacemaker Implantation and Complication Costs **eFigure 2.** Illustration of the Structure of the Markov Simulation Model for a Patient With Postoperative PPM Implantation After CHS **eFigure 3.** Distribution of Congenital Heart Disease Severity in Cost Analysis Patient Cohorts
eReferences

Supplement 2.
Data Sharing Statement

## References

[1] Castañeda A. Congenital heart disease: a surgical-historical perspective. Ann Thorac Surg. 2005;79(6):S2217-S2220. doi:10.1016/j.athoracsur.2005.03.031 15919255

[2] Aquilina O. A brief history of cardiac pacing. Images Paediatr Cardiol. 2006;8(2):17-81. 22368662 PMC3232561

[3] Liberman L, Silver ES, Chai PJ, Anderson BR. Incidence and characteristics of heart block after heart surgery in pediatric patients: a multicenter study. J Thorac Cardiovasc Surg. 2016;152(1):197-202. doi:10.1016/j.jtcvs.2016.03.081 27167020

[4] Romer AJ, Tabbutt S, Etheridge SP, . Atrioventricular block after congenital heart surgery: analysis from the Pediatric Cardiac Critical Care Consortium. J Thorac Cardiovasc Surg. 2019;157(3):1168-1177.e2. doi:10.1016/j.jtcvs.2018.09.142 30917883

[5] Gregoratos G, Abrams J, Epstein AE, . ACC/AHA/NASPE 2002 guideline update for implantation of cardiac pacemakers and antiarrhythmia devices–summary article: a report of the American College of Cardiology/American Heart Association Task Force on Practice Guidelines (ACC/AHA/NASPE Committee to Update the 1998 Pacemaker Guidelines). J Am Coll Cardiol. 2002;40(9):1703-1719. doi:10.1016/S0735-1097(02)02528-7 12427427

[6] McLeod KA. Cardiac pacing in infants and children. Heart. 2010;96(18):1502-1508. doi:10.1136/hrt.2009.173328 20813732

[7] Silka MJ, Bar-Cohen Y. Pacemakers and implantable cardioverter-defibrillators in pediatric patients. Heart Rhythm. 2006;3(11):1360-1366. doi:10.1016/j.hrthm.2006.02.009 17074646

[8] Singh HR, Batra AS, Balaji S. Pacing in children. Ann Pediatr Cardiol. 2013;6(1):46-51. doi:10.4103/0974-2069.107234 23626436 PMC3634247

[9] Fortescue EB, Berul CI, Cecchin F, Walsh EP, Triedman JK, Alexander ME. Patient, procedural, and hardware factors associated with pacemaker lead failures in pediatrics and congenital heart disease. Heart Rhythm. 2004;1(2):150-159. doi:10.1016/j.hrthm.2004.02.020 15851146

[10] Lau KC, William Gaynor J, Fuller SM, Smoots KA, Shah MJ. Long-term atrial and ventricular epicardial pacemaker lead survival after cardiac operations in pediatric patients with congenital heart disease. Heart Rhythm. 2015;12(3):566-573. doi:10.1016/j.hrthm.2014.12.001 25484105

[11] Madhavan M, Mulpuru SK, McLeod CJ, Cha YM, Friedman PA. Advances and future directions in cardiac pacemakers: part 2 of a 2-part series. J Am Coll Cardiol. 2017;69(2):211-235. doi:10.1016/j.jacc.2016.10.064 28081830

[12] Mulpuru SK, Madhavan M, McLeod CJ, Cha YM, Friedman PA. Cardiac pacemakers: function, troubleshooting, and management: part 1 of a 2-part series. J Am Coll Cardiol. 2017;69(2):189-210. doi:10.1016/j.jacc.2016.10.061 28081829

[13] Czosek RJ, Meganathan K, Anderson JB, Knilans TK, Marino BS, Heaton PC. Cardiac rhythm devices in the pediatric population: utilization and complications. Heart Rhythm. 2012;9(2):199-208. doi:10.1016/j.hrthm.2011.09.004 21907171

[14] Wei H, Roscigno CI, Hanson CC, Swanson KM. Families of children with congenital heart disease: a literature review. Heart Lung. 2015;44(6):494-511. doi:10.1016/j.hrtlng.2015.08.005 26404115

[15] Edelson JB, Rossano JW, Griffis H, . Emergency department visits by children with congenital heart disease. J Am Coll Cardiol. 2018;72(15):1817-1825. doi:10.1016/j.jacc.2018.07.055 30286926

[16] Gutierrez-Colina AM, Eaton C, Cheng P, . Perceived self-competence, psychosocial adjustment, and quality of life in pediatric patients with pacemakers. J Dev Behav Pediatr. 2014;35(6):360-366. doi:10.1097/DBP.0000000000000073 25007058

[17] Webster G, Panek KA, Labella M, . Psychiatric functioning and quality of life in young patients with cardiac rhythm devices. Pediatrics. 2014;133(4):e964-e972. doi:10.1542/peds.2013-1394 24664095 PMC3966499

[18] Sachse FB, Johnson J, Cottle B, Mondal A, Hitchcock R, Kaza AK. Toward detection of conduction tissue during cardiac surgery: light at the end of the tunnel? Heart Rhythm. 2020;17(12):2200-2207. doi:10.1016/j.hrthm.2020.07.008 32659372 PMC7704810

[19] Panni RZ, Ashfaq A, Amanullah MM. Earlier surgical intervention in congenital heart disease results in better outcome and resource utilization. BMC Health Serv Res. 2011;11:353. doi:10.1186/1472-6963-11-353 22206493 PMC3277492

[20] Sonnenberg FA, Beck JR. Markov models in medical decision making: a practical guide. Med Decis Making. 1993;13(4):322-338. doi:10.1177/0272989X9301300409 8246705

[21] Income, poverty and health insurance coverage in the United States: 2017. US Census Bureau; 2018. Accessed June 15, 2019. https://www.census.gov/newsroom/press-releases/2018/income-poverty.html

[22] Erikssen G, Liestøl K, Seem E, . Achievements in congenital heart defect surgery: a prospective, 40-year study of 7038 patients. Circulation. 2015;131(4):337-346. doi:10.1161/CIRCULATIONAHA.114.012033 25538230

[23] Raissadati A, Nieminen H, Jokinen E, Sairanen H. Progress in late results among pediatric cardiac surgery patients: a population-based 6-decade study with 98% follow-up. Circulation. 2015;131(4):347-353. doi:10.1161/CIRCULATIONAHA.114.011190 25538229

[24] Freedom RM, Lock J, Bricker JT. Pediatric cardiology and cardiovascular surgery: 1950-2000. Circulation. 2000;102(20)(suppl 4):IV58-IV68. 11080133 10.1161/01.cir.102.suppl_4.iv-58

[25] DiFazio R, Vessey J. Nonmedical out-of-pocket expenses: a hidden cost of hospitalization. J Pediatr Nurs. 2011;26(1):78-84. doi:10.1016/j.pedn.2010.01.010 21256415

[26] Czosek RJ, Bonney WJ, Cassedy A, . Impact of cardiac devices on the quality of life in pediatric patients. Circ Arrhythm Electrophysiol. 2012;5(6):1064-1072. doi:10.1161/CIRCEP.112.973032 23212181

