# Supplementary material for: Cost of Pacing in Pediatric Patients With Postoperative Heart Block After Congenital Heart Surgery
Source: JAMA Netw Open. 2023 Nov 3;6(11):e2341174. doi: 10.1001/jamanetworkopen.2023.41174 (PMC10625035; doi:10.1001/jamanetworkopen.2023.41174)
Supplement: Supplement 1. — eMethods eTable 1. Medical Procedure Costs eTable 2. Cost Considerations for Accompanying a Patient to the Hospital eFigure 1. Permanent Pacemaker Implantation and Complication Costs eFigure 2. Illustration of the Structure of the Markov Simulation Model for a Patient With Postoperative PPM Implantation After CHS eFigure 3. Distribution of Congenital Heart Disease Severity in Cost Analysis Patient Cohorts eReferences [file jamanetwopen-e2341174-s001.pdf]

Supplemental Online Content

Mondal A, Yoo M, Tuttle S, et al. Cost of pacing in pediatric patients with postoperative heart block after congenital heart surgery. *JAMA Netw Open*. 2023;6(11):e2341174. doi:10.1001/jamanetworkopen.2023.41174

eMethods

eTable 1. Medical Procedure Costs

eTable 2. Cost Considerations for Accompanying a Patient to the Hospital

eFigure 1. Permanent Pacemaker Implantation and Complication Costs

eFigure 2. Illustration of the structure of the Markov Simulation Model for a Patient With Postoperative PPM Implantation After CHS

eFigure 3. Distribution of Congenital Heart Disease Severity in Cost Analysis Patient Cohorts

eReferences

This supplemental material has been provided by the authors to give readers additional information about their work.

© 2023 Mondal A et al. *JAMA Network Open.*

eMethods

The analysis in this study was performed using MATLAB 2019b (MathWorks Inc., Natick, MA), TreeAge Pro 2018, version 2.1 (TreeAge Software, Williamstown, MA), and Excel 365 version 1912 (Microsoft Corp., Redmond, WA).

Trends in congenital heart surgeries

CHS procedure codes were used to identify surgical procedures between 1960 and 2018. Codes associated with follow-up or support surgeries performed following CHS were excluded. The surgery date, patient age during surgery, and the attending surgeon were collected from this group. Each CHS was binned to the year of surgery (1960-2018) and grouped based on patient age (age less than 4 years and greater than equal to 4 years). Generalized linear model with Poisson distribution was utilized to assess the annual trend.

Trends in postoperative PPM implantation

Patients identified with postoperative PPM implantation were grouped based on patient age at PPM implantation (age less than 4 years and greater than equal to 4 years) and year of surgery (1960-2018). The number of such implantations per CHS performed for the respective years was calculated. Generalized linear model with Poisson distribution was utilized to assess the annual trend.

Major and minor procedure costs

Direct medical costs of major PPM-related events were computed from hospital charges of selected representative events between 2010-2018 (eFigure 1). All charges were inflated to 2018 costs using the consumer price index.[1] All other relevant procedure costs were collected from the 2018 Boston Children's Hospital chargemaster.[2]

Determination of LOS for major and minor hospital events

LOS for minor outpatient procedures and consultation visits were assumed to be 3 hours.
LOS for major procedures was determined as follows:

Pacemaker implantation

1. Calculate the difference between the discharge date and index surgery date.
2. If the difference between discharge date and index surgery date <=10 days
   a. Then LOS was the number of days to the discharge date from the index surgery date.
3. If the difference between the discharge date and index surgery date >10 days
   a. Then LOS was the number of days between PPM implantation and the next procedure (surgery or cath).
4. If the calculated LOS was greater than 30 days
   a. Then LOS value was discarded and not considered in the economic evaluation.

Pacemaker malfunction: generator and lead replacement

1. If the procedure was Outpatient,
   a. Then LOS = 1 day
2. If the procedure was Inpatient
   a. Then calculate the difference between the discharge date and the admission date of the malfunction event.
3. If the difference between discharge date and admission date <=7 days
   a. Then LOS was the number of days to the discharge date from the admission date.
4. If the difference between the discharge date and admission date >7 days
   a. Then LOS was the number of days between PPM implantation and the next procedure (surgery or Cath).
5. If the calculated LOS was greater than 30 days
   a. Then LOS value was discarded and not considered in the economic evaluation

Pacemaker infection

1. If the procedure was Outpatient,
   a. Then LOS = 3 hours
2. If the procedure was Inpatient

© 2023 Mondal A et al. *JAMA Network Open.*

a. Then LOS was the difference between the discharge date and the admission date of the infection event.

Pacemaker replacement: battery depletion or generator change

1. If the procedure was Outpatient,
   a. Then LOS = 3 hours
2. If the procedure was Inpatient
   a. Then LOS was the difference between the discharge date and the admission date of the pacemaker replacement event.

EP-Cath procedure

1. If the procedure was Outpatient,
   a. Then LOS = 1 day
2. If the procedure was Inpatient
   a. Then LOS was the difference between the discharge date and the admission date of the EP-Cath event.

Markov model input parameters

The movement of hypothetical patients through the model was governed by probability input parameters. Adverse event probabilities included each complication and event probabilities for healthcare visits were estimated using the selected patient procedure data. These were patients who had PPM implanted at an age less than 4 years and had at least 6 months of follow-up data. When multiple healthcare utilization for the same event was observed, those that happened more than 7 days apart were considered separate events. Both direct and indirect costs included those related to the treatment of concomitant complications, as well as the cost for the index PPM implantation for each patient from the time of the first implantation until the end of the 20-year time horizon (eTables 1 and 2). The indirect costs for each event were estimated based on the time off of work and US average household income, costs for accommodation, trips to/from the clinic, parking, meals and incidentals. All costs were adjusted to 2018 US dollars.[1]

Markov model sensitivity analysis

Point estimates for each input parameter value were used in the base-case analysis. We also performed probabilistic sensitivity analysis (PSA) to assess the impact of uncertainty in all input parameters simultaneously using 1,000,000 (1,000 trials of 1,000 hypothetical patients each) simulated patients. In PSA trials, parameter values were based on random draws from a distribution. Event probabilities were assumed to follow a beta distribution and cost estimates were assumed to follow a gamma distribution.

Cost estimation of clinical courses with and without complication using follow-up data

Direct medical costs for each patient for each year were calculated using estimated event costs (eTable 1) and aggregated to the respective 1-year periods from PPM implantation. Once costs from all patients were binned, the average cost for each year was calculated. Cumulative costs starting from years 1 to 20 were calculated and plotted. Indirect costs or the cost of accompanying a patient for medical care were calculated utilizing LOS for each visit. The cost of lost productivity, accommodation, transportation, and food was included as indirect costs. eTable 2 lists the costs considered for indirect cost calculation. Accommodation costs were considered only for visits with LOS >1 day. The cost of transportation using a personally owned vehicle (POV) per hospital visit was calculated utilizing the national average distance to the nearest pediatric cardiology subspeciality[3] and POV mileage rate (eTable 2, row 4). Like direct costs, costs for all patients were aggregated to the respective year of the event and the average indirect cost for each year and cumulative 20-year cost were calculated.

Statistical Analysis

Linear regression analysis (LR) was performed to determine the dependence of complication cost on LOS and to extrapolate the 20-year costs. R^2 and P values were calculated and used to determine dependence and statistical significance, respectively.

Poisson generalized linear model (GLM) was utilized to assess annual trends in CHS, surgical team size and PPM implantation. The model used the following log-link function:-

$$\log y = \beta x + 1$$

© 2023 Mondal A et al. *JAMA Network Open.*

eTable 1. Medical Procedure Costs

Medical procedure/event	Cost (2018 USD)	Source
**Pacemaker implantation**	$27,460 + 6,197×LOS	Calculated from 2010-18 patient hospital billing data
**Pacemaker malfunction requiring generator and lead replacement**	$31,450 + 6,740×LOS	Calculated from 2010-18 patient hospital billing data
**Pacemaker infection**	$2,079 + 7,065×LOS	Calculated from 2010-18 patient hospital billing data
**Generator change/ battery depletion**	$41,797 ± 11,050	Calculated from 2010-18 patient hospital billing data
**In-clinic consults/visit**	$219	BCH chargemaster (2018)
**In-clinic device check**	$463	BCH chargemaster (2018)
**ECG**	$289	BCH chargemaster (2018)
**Holter**	$963	BCH chargemaster (2018)
**EP-Cath procedure**	$5,894	BCH chargemaster (2018)
**Cardiac rehab**	$311	BCH chargemaster (2018)

LOS – Length of stay

© 2023 Mondal A et al. *JAMA Network Open.*

eTable 2. Cost Considerations for Accompanying a Patient to the Hospital

	Cost (2018 USD)	Source
**US household income**	$61,372 per annum	www.census.gov
**Accommodation**	$248 per night	www.gsa.gov
**POV* mileage rate**	$0.445 per mile	www.gsa.gov
**Average hospital trip cost using POV**	$15.4 per trip	Turner et al. 2020[3] and www.gsa.gov
**Parking**	$10 per day	www.childrenshospital.org
**Meals and incidentals**	$69 per day	www.gsa.gov

*Personally Owned Vehicle

© 2023 Mondal A et al. *JAMA Network Open.*

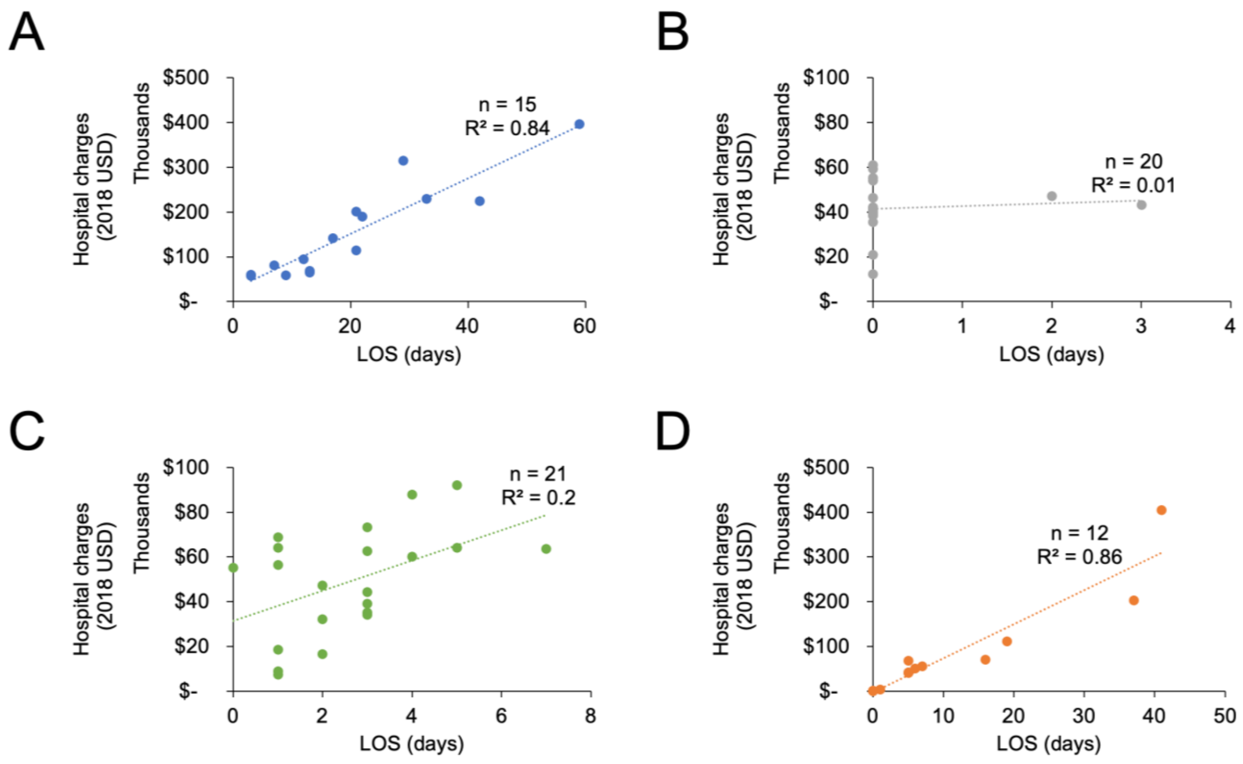

eFigure 1. Permanent Pacemaker Implantation and Complication Costs. Plots showing the total hospital charge for pacemaker related major procedures vs length of stay (LOS). (A) PPM implantation cost vs LOS (RL: $R^2 = 0.838$, $P < 0.001$) from 15 patients after congenital heart surgeries till patient discharge. (B) PPM-generator change costs vs LOS (RL: $R^2 = 0.007$, $P = 0.721$) of hospital events from 20 patients. (C) PPM malfunction requiring lead and generator replacement event costs vs LOS (RL: $R^2 = 0.23$, $P < 0.05$) from 21 patients. (D) Pacemaker infection event costs vs LOS from 12 patients ($R^2 = 0.864$, $P < 0.001$). LOS of 0 indicates outpatient procedure. LOS – Length of stay.

© 2023 Mondal A et al. *JAMA Network Open.*

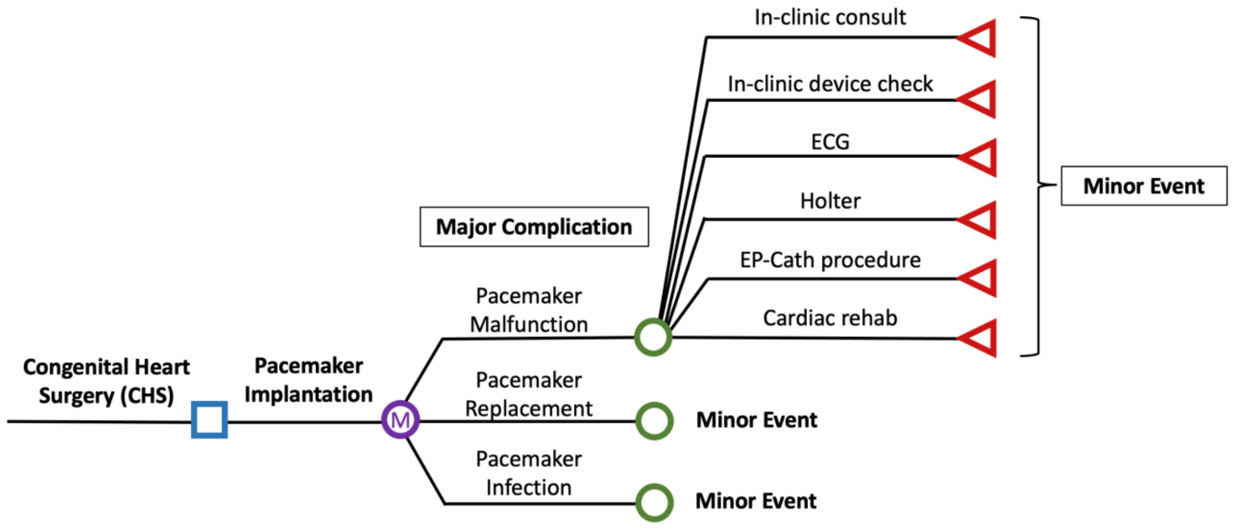

eFigure 2. Illustration of the Structure of the Markov Simulation Model for a Patient With Postoperative PPM Implantation After CHS.

© 2023 Mondal A et al. *JAMA Network Open.*

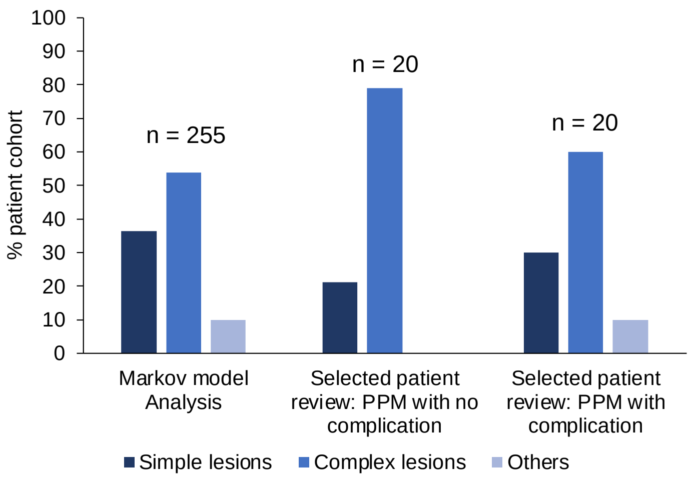

eFigure 3. Distribution of Congenital Heart Disease Severity in Cost Analysis Patient Cohorts. Simple lesions: ASD, VSD, AVC, TOF. Complex lesions: DORV, L-TGA, D-TGA, HLHS, DIRV, DILV, TAPVR, TA, IAA. Others: CCHB, Sinus bradycardia, Tachycardia, PDA, Ebstein's anamoly, Shone's syndrome, TV surgery, AV surgery, MV surgery, RVOTO, Cor triatriatum sinister.

© 2023 Mondal A et al. *JAMA Network Open.*

eReferences

1. Consumer Price Index Boston-Cambridge-Newton, New England Information Office: U.S. Bureau of Labor Statistics. https://www.bls.gov/regions/new-england/data/consumerpriceindex_boston_table.htm

2. Boston Children's Hospital Standard Charge Information. https://www.childrenshospital.org/patient-resources/financial-and-billing-matters/hospital-services-and-charges

3. Turner A, Ricketts T, Leslie LK. Comparison of Number and Geographic Distribution of Pediatric Subspecialists and Patient Proximity to Specialized Care in the US Between 2003 and 2019. *JAMA Pediatr*. Sep 1 2020;174(9):852-860. doi:10.1001/jamapediatrics.2020.1124

© 2023 Mondal A et al. *JAMA Network Open.*
