# Supplementary material for: Cost of Pacing in Pediatric Patients With Postoperative Heart Block After Congenital Heart Surgery
Source: JAMA Netw Open. 2023 Nov 3;6(11):e2341174. doi: 10.1001/jamanetworkopen.2023.41174 (PMC10625035; doi:10.1001/jamanetworkopen.2023.41174)
Supplement: Supplement 2. — Data Sharing Statement [file jamanetwopen-e2341174-s002.pdf]

Data Sharing Statement

Mondal. Cost of Pacing in Pediatric Patients With Postoperative Heart Block After Congenital Heart Surgery. *JAMA Netw Open*. Published November 02, 2023.
doi:10.1001/jamanetworkopen.2023.41174

Data
Data available: Yes
Data types: Data (not involving human participants), Data dictionary
How to access data: Data will be made available by contacting the corresponding author, Dr. Aditya K. Kaza at aditya.kaza@cardio.chboston.org
When available: With publication

Supporting Documents
Document types: None

Additional Information
Who can access the data: Data will be made available to anyone requesting the data.
Types of analyses: For any purpose
Mechanisms of data availability: With signed data access agreement
